# Influence of Single Nucleotide Polymorphisms on Rifampin Pharmacokinetics in Tuberculosis Patients

**DOI:** 10.3390/antibiotics9060307

**Published:** 2020-06-08

**Authors:** Levin Thomas, Sonal Sekhar Miraj, Mallayasamy Surulivelrajan, Muralidhar Varma, Chidananda S. V. Sanju, Mahadev Rao

**Affiliations:** 1 Department of Pharmacy Practice, Manipal College of Pharmaceutical Sciences, Manipal Academy of Higher Education, Manipal, Karnataka 576104, India; levin.thomas@learner.manipal.edu (L.T.); sonal.sekhar@manipal.edu (S.S.M.); msv.rajan@manipal.edu (M.S.); 2 Department of Infectious Diseases, Kasturba Medical College, Manipal Academy of Higher Education, Manipal, Karnataka 576104, India; muralidhar.varma@manipal.edu; 3 District Tuberculosis Control Office, Ajjarakad, Udupi, Karnataka 576001, India; dtokaudu@rntcp.org

**Keywords:** tuberculosis, rifampin, single nucleotide polymorphisms, *SLCO1B1*, pharmacokinetics

## Abstract

Rifampin (RF) is metabolized in the liver into an active metabolite 25-desacetylrifampin and excreted almost equally via biliary and renal routes. Various influx and efflux transporters influence RF disposition during hepatic uptake and biliary excretion. Evidence has also shown that Vitamin D deficiency (VDD) and Vitamin D receptor (VDR) polymorphisms are associated with tuberculosis (TB). Hence, genetic polymorphisms of metabolizing enzymes, drug transporters and/or their transcriptional regulators and VDR and its pathway regulators may affect the pharmacokinetics of RF. In this narrative review, we aim to identify literature that has explored the influence of single nucleotide polymorphisms (SNPs) of genes encoding drug transporters and their transcriptional regulators (SLCO1B1, ABCB1, PXR and CAR), metabolizing enzymes (CES1, CES2 and AADAC) and VDR and its pathway regulators (VDR, CYP27B1 and CYP24A1) on plasma RF concentrations in TB patients on antitubercular therapy. Available reports to date have shown that there is a lack of any association of *ABCB1*, *PXR*, *CAR*, *CES1* and *AADAC* genetic variants with plasma concentrations of RF. Further evidence is required from a more comprehensive exploration of the association of *SLCO1B1*, *CES2* and Vitamin D pathway gene variants with RF pharmacokinetics in distinct ethnic groups and a larger population to reach conclusive information.

## 1. Introduction

Rifampin (RF) was introduced as a part of the combinational chemotherapy regimen for tuberculosis (TB) during the 1960s. This has revolutionized TB treatment by reducing the duration of antitubercular therapy (ATT) and improving the cure rates [1,2]. The antimicrobial effect of RF on *Mycobacterium tuberculosis* and the development of RF resistance is concentration-dependent [3,4]. RF exhibits antimycobacterial action by arresting the DNA-directed RNA synthesis of *Mycobacterium tuberculosis* through interaction with the β subunit of RNA polymerase (RNAP) [5,6]. The primary mechanism of RF resistance is due to the mutations in the *rpoB* gene that encode for the β-subunit of RNA polymerase. The most common mutations in the *rpoB* gene are found in the *rpoB* 531, *rpoB* 526 and *rpoB* 516 codons of the RF resistance determining region (RRDR) [7,8]. Recent evidence has shown that higher doses of RF from the currently recommended dosage regimens resulted in better treatment outcomes in TB patients [9,10]. A recent comprehensive meta-analysis reported a wide range of interstudy heterogeneity in RF pharmacokinetic parameter estimates. Many variables such as HIV, TB and diabetes status, drug combinations, duration of therapy and dosing frequency could not explain the heterogeneity in the pharmacokinetics of RF. An increase in RF dose from the common weight-based dosing category of 8–12 mg/kg to at least 25 mg/kg was required to achieve plasma pharmacokinetic-pharmacodynamic (PK/PD) targets [11]. Single nucleotide polymorphisms (SNPs) represent the most common type of genetic polymorphism in humans [12]. Multiple studies have reported the association of various genetic polymorphisms with significant variances in plasma RF levels in TB patients. This provides us with an exciting opportunity to review for assessing the potential impact of SNPs as an important driver for plasma RF exposure variability in TB patients.

RF is metabolized in the liver into an active metabolite 25-desacetylrifampin and excreted almost equally via biliary and renal routes [13]. B-esterase and Arylacetamide deacetylase (AADAC) enzymes have been reported to catalyze the deacetylation of RF to 25-deacetylrifampin [14,15]. Membrane drug transporters are recognized to be important determinants of absorption, distribution, metabolism and excretion (ADME) of drugs and consequently influence their pharmacokinetic (PK), therapeutic efficacy and safety profiles. Solute carrier (SLC) transporters and the adenosine triphosphate (ATP)-binding cassette (ABC) transporters represent two superfamilies of membrane drug transporters. They are primarily involved in the in and out transport of drugs across tissues and cells in the human body. The SLC and ABC superfamily account for about 400 membrane transporters, out of which around 32 are clinically relevant [16,17]. Pregnane X receptor (PXR) and constitutive androstane receptor (CAR) are nuclear hormone receptors that are involved in the transcriptional regulation of various drug-metabolizing enzymes and transporters [18]. Multiple studies have revealed the potential role of PXR and CAR in the transcriptional regulation of SLC and ABC proteins [19,20,21]. RF disposition is influenced by sinusoidal influx transporter SLCO1B1 and efflux transporter ABCB1 during hepatic uptake and biliary excretion, respectively [22,23,24].

Vitamin D regulates gene transcription by binding to Vitamin D Receptor (VDR). The 427 amino acid VDR is encoded by the *VDR* gene [25]. Vitamin D is involved in the modulation of innate and adaptive immune responses through the mediation of multiple genes. These genes regulated by the transcription factor VDR encode for proteins that relate to acute response to infection, general functions in infection and for autoimmune responses [26]. The degree of immune responses elicited is associated with the circulating levels of Vitamin D [27]. Vitamin D deficiency (VDD) and *VDR* gene polymorphisms are associated with an increased risk for the development of TB [28]. VDR has been reported to induce the expression of *SLCO1B1* [29]. Furthermore, RF can also result in the reduction of Vitamin D levels by increasing its clearance through the agonist and inducing action on PXR and CYP3A4, respectively [30,31]. Hence, the genetic polymorphisms of these metabolizing enzymes, drug transporters and/or their transcriptional regulators and *VDR* gene and its pathway regulators may influence the RF pharmacokinetics.

**Figure 1:**
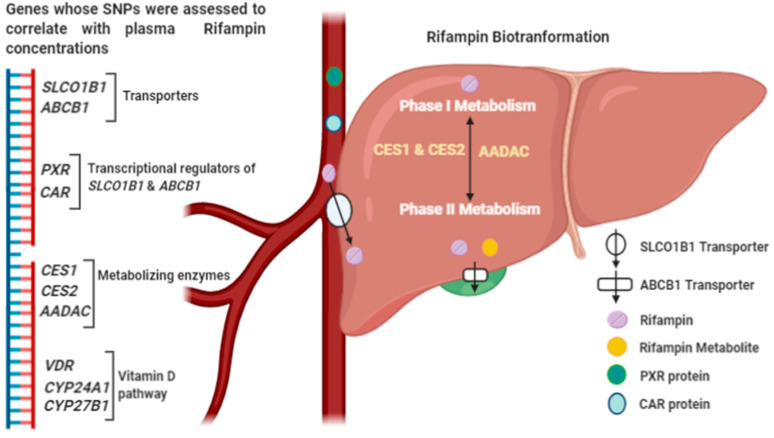
Schematic diagram representing. (1) The genes whose SNPs were assessed with plasma RF concentrations in the review and (2) RF biotransformation.

Relevant studies were searched in databases like PubMed, MEDLINE, EMBASE, Web of Science and Google Scholar. The following Medical Subject Headings (MeSH) words were used as part of our search strategy: antitubercular agents, antitubercular drugs, rifampin, rifampicin, genetic polymorphism, genetic susceptibility, pharmacogenetics, pharmacogenomics, genetic association study, genetic association analysis, tuberculosis, single nucleotide polymorphisms, pharmacokinetics, population pharmacokinetics, *SLCO1B1*, *ABCB1*, *PXR, CAR*, carboxylesterase 1 (*CES1*), carboxylesterase 2 (*CES2*), *AADAC* and *VDR*. The scope of the review is limited to studies that recruited TB patients, regardless of age and HIV status who were either already established on ATT or commencing treatment. Association between at least one genetic variant and RF pharmacokinetic outcome was assessed (Figure 1). Studies without any formal evaluation of genotype effects for RF exposures were excluded. From the reference lists of the articles, we extracted additional literature relevant to the topic. Only publications in the English language were considered for this review.

## 2. SLCO1B1

The organic anion transporting polypeptide 1B1 (OATP1B1) is a 691 amino acid protein expressed predominantly on the basolateral (sinusoidal) membrane of hepatocytes. It is encoded by the solute carrier organic anion transporter family member 1B1 (*SLCO1B1)* gene (spans 15 exons) located on chromosome 12. OATP1B1 is one of the major membrane influx transporters that regulate the active hepatic uptake of substrates from the bloodstream into the hepatocyte [16,32]. RF is a strong substrate of the OATP1B1 transporter protein [33,34]. Around 190 genomic variants with minor allele frequency higher than 5% were identified with the *SLCO1B1* gene. Among these variants, rs4149056 and rs2306283 have been commonly identified and well-characterized [35].

The missense SNP rs4149056 located in exon 5 (also known as c.521T>C; with T allele defined as the wild-type allele and the C allele as a variant) causes a change of amino acid from valine to alanine at residue 174. This variant is reported to have reduced expression and activity of *SLCO1B1* in vitro and in vivo. Hence, drugs that are substrates for OATP1B1 with c.521T>C may tend to have elevated plasma concentrations due to reduced uptake/transporter activity [36,37]. Allegra et al. have reported higher plasma RF concentrations in TB patients with *SLCO1B1* rs4149056 polymorphism. Multivariate linear regression analysis revealed that *SLCO1B1* rs4149056 genotype was found to be a positive predictive factor for increased plasma RF trough concentration (C_trough_, *p* = 0.048, β = 0.345, 95% CI [6.458–1313.556]) and maximum concentration (C_max_, *p* = 0.019, β = 0.432, 95% CI [452.896–4571.730] at second week of ATT [38]. The frequency of *SLCO1B1* rs4149056 genotype was reported to be 28.3%, 5.7%, 14.9% and 14.8% in Amerindian, African descent, Mulatto and Caucasian descent ethnic groups, respectively [39]. Mwinyi et al. reported a frequency of 15% and 12.2% in German and Turkish populations, respectively, whereas 15% prevalence was reported among the UK population for *SLCO1B1* rs4149056 genotype [40,41].

rs2306283 (c.388A>G) is a missense SNP located in the exon 4 of the *SLCO1B1* gene that causes a change of amino acid from asparagine to aspartic acid at amino acid position 130. The functional consequences of this variant reported by different in vitro and in vivo studies have yielded conflicting results and may be substrate-specific [36,42]. Dompreh et al. had reported that the *SLCO1B1* rs2306283 polymorphism was associated with lower RF concentration in the pediatric TB population. Two patients (1.8%) with the *SLCO1B1* *1b homozygous variant (AA genotype) had significantly lower RF C_max_ (1.81 (0.81–2.80) μg/mL) and area under the time-concentration curve from 0 to 8 h (9.33 (2.35–16.31) μg*h/mL) and higher apparent oral clearance (44.54 (15.38–73.69) L/h) and apparent volume of distribution (109.23 (54.86–163.59) L) than did those with the wild type (GG genotype) in a pairwise analysis [43]. However, other studies have reported higher frequencies of the *SLCO1B1* *1b homozygous variant (AA genotype) in Chilean (18.6%), Macedonian (33.1%) and Albanian (30.8%) population [44,45].

Chigutsa et al. and Gengiah et al. reported a high prevalence of *SLCO1B1* rs4149032 (g.38664C>T), which is an intron 2 haplotype tagging SNP (tSNP). *SLCO1B1* rs4149032 polymorphism was found to be associated with lower RF exposures in the African population suggesting the need for increasing the RF dose [46,47]. The functional consequences of *SLCO1B1* rs4149032 on gene expression and on transporter activity are not yet known. Chigutsa et al. reported an allele frequency of 70% for the *SLCO1B1* rs4149032 polymorphism in the South African pulmonary TB (PTB) patients. Patients who were heterozygous and homozygous for the rs4149032 polymorphism in this population had reductions in RF bioavailability by 18% and 28%, respectively. Simulations showed that *SLCO1B1* rs41490932 carriers had a predicted reduction in C_max_ of < 8 mg/L and an increase in the daily rifampin dose by 150 mg in the PTB patients in these population would help in achieving plasma concentrations similar to those of wild-type individuals [46]. Gengiah et al. reported an allele frequency of 76% for the *SLCO1B1* rs4149032 polymorphism in the TB-HIV coinfected patients in South Africa. The median (IQR) RF concentrations at 2.5 h postdose were 3.4 (2.7–4.7) μg/mL, 3.7 (2.8–5.0) μg/mL and 5.3 (3.8–6.7) μg/mL for homozygous variant, heterozygous variant and wild type carriers of *SLCO1B1* rs4149032 polymorphism, respectively, which was well below the recommended target range of 8 to 24 μg/mL [47]. Mukonzo et al. reported an allelic frequency of 66% for the *SLCO1B1* rs4149032 polymorphism in the Ugandan population [48].

Lower RF exposures were reported with *SLCO1B1* rs11045819 polymorphism in a study conducted by Weiner et al. [49]. *SLCO1B1* rs11045819 (c.463 C>A) polymorphism is a missense variant, present on the exon 4 of the *SLCO1B1* that cause a change of amino acid from proline to threonine at amino acid position 155 [49]. *SLCO1B1* rs11045819 polymorphism was found to reduce the systemic exposure of the substrate for OATP1B1 transporter [50]. Weiner et al. reported the prevalence of *SLCO1B1* rs11045819 polymorphism as 19% (*n* = 7) in African TB patients, 11% (*n* = 4) in TB patients of US and Spain and 25% (*n* = 4) among the healthy US population (controls). Patients with the *SLCO1B1* rs11045819 variant allele (CA) had 42% lower RF exposure (25.6 μg*h/mL), 34% lower peak concentration levels (5 μg/mL) and 63% greater apparent oral clearance (22 L/h) compared to the wild type allele (CC) [49].

However, recent studies from the African population have not found any association with *SLCO1B1* polymorphisms and RF exposures among TB patients [48,51,52]. Similarly, studies conducted by Ramesh et al. and Jeremiah et al. in the Indian and Tanzanian population, respectively, did not report any association of *SLCO1B1* polymorphisms with plasma RF exposures (Table 1) [53,54]. The association of *SLCO1B1* rs4149056, rs2306283, rs4149032 and rs11045819 polymorphisms with RF pharmacokinetics reported in certain studies were not replicated in other studies that can be attributed due to multiple factors such as lower sample population, ethnic variations, variations in the criteria and timings of sample collection, analytical variations and interindividual factors such as variations in body weight and medication adherence. Therefore, additional studies are warranted to characterize the functional consequences of *SLCO1B1* rs4149056, rs2306283, rs4149032 and rs11045819 polymorphism on RF pharmacokinetics in other ethnic groups.

## 3. ABCB1

*ABCB1* (or *MDR1*) gene is located on chromosome 7 and consists of 29 exons in a genomic region spanning 251.3 kb. It is one of many ABC genes that encode for the 1280 amino acid ABCB1 transporter protein (P-glycoprotein). P-glycoprotein (Pgp) is a multidomain integral membrane protein that utilizes the energy generated from the ATP hydrolysis to translocate solutes or ions from intracellular to extracellular membranes (efflux pump) in eukaryotes [56,57,58]. RF is a substrate of the Pgp efflux pump [59]. rs1128503, rs2032582 and rs1045642 are the most commonly found SNPs in the *ABCB1* gene [60]. rs1128503 and rs1045642 are synonymous mutations, whereas rs2032582 is a missense mutation [61]. None of the studies were able to infer any association between *ABCB1* polymorphisms and RF pharmacokinetics (Table 2). These studies have explored the association of only a limited number of *ABCB1* polymorphisms with the RF exposures. There are about 8643 single nucleotide variants (SNV) reported for the *ABCB1* gene. The functional consequences of rare *ABCB1* variants that may have a significant effect on drug pharmacokinetics have not been largely elucidated [58]. Hence, additional studies with other genetic variants are required to establish the impact of *ABCB1* polymorphisms with the RF exposure.

## 4. PXR and CAR

PXR and the CAR are members of the group I of the subfamily 1 of nuclear receptors (NRs) that are involved in regulating the transcription of a wide range of drug-metabolizing enzymes and drug transporters genes [62,63]. RF is a substrate for SLCO1B1 and ABCB1 protein and the transcription of genes encoding these proteins are regulated by the *PXR* and *CAR*. Few studies have explored the possibility of association of the SNPs of these genes with the plasma RF levels. The *PXR* (or *NR1I2*) gene located on chromosome 3 and consisting of 9 exons encodes for the PXR [64]. rs2472677 and rs1523130 variants are present in the intron 1 and 5′UTR regions of the *PXR* gene, respectively. These regions represent the transcription factor binding sites of *PXR* regulatory regions [65,66]. The *CAR* (or *NR1I3*) gene located on chromosome 1 and consisting of 9 exons encodes for the CAR [67,68]. The rs2307424 variant is due to a synonymous substitution (c.540 C>T) in the *CAR* gene [69]. None of these SNPs in *PXR* and *CAR* affected RF exposures (Table 3 and Table 4).

## 5. CES1 and CES2

RF is primarily metabolized to 25-desacetylrifampin by B-esterase [70]. B-esterases family comprises CES, acetylcholinesterase and butyrylcholinesterase enzymes [14]. Among these enzymes, CES exhibits broad substrate specificity and is involved in the metabolism of a wide range of endobiotic and xenobiotic compounds by hydrolyzing ester, thioester, amide and carbamate linkages. Human CES1 and human CES2 encoded by *CES1* and *CES2* gene, respectively, represent the two major isoenzymes of CES that are expressed in the liver [71]. Over the past decade, several *CES1* and *CES2* functional genetic variants associated with significant variations to various drug therapy responses have been reported. Hence, assessing the genetic polymorphisms of these genes with the pharmacokinetics of the substrate drugs becomes relevant [72]. The *CES1* and *CES2* genes are located on chromosome 16 and consist of 14 and 12 exons, respectively [73].

Sloan et al. reported that the rs12149368 variant present on the exon 1 (5’UTR) region of the *CES1* gene does not affect the plasma RF concentration (Table 5) [55]. Song et al. evaluated 10 SNPs: c.-2548C>T and c.-2263A>G variants in the promoter region, c.269-965A>G, c.474-152T>C, c.615 + 120G>A, c.1612 + 136G>A and c.1613-87G>A variants of the intron regions and c.1872*69A>G, c.1872*302_304delGAA, c.1872*445C>T variants of the 3′UTR regions of the *CES2* gene with the RF levels. Increased plasma RF concentrations in TB patients were associated with the *CES2* c.-22263A>G (g.738A>G) variant. The allelic frequencies for this variant were reported to be 0.33 in TB patients and 0.31 in controls and plasma RF concentrations were 8.9 ± 2.9 mg/L, 10.5 ± 3.1 mg/L and 13.9 ± 7.4 mg/L in homozygotes carrying major allele, heterozygotes and homozygotes carrying minor allele, respectively. Results of luciferase reporter analysis revealed that the change from A to G in *CES2* c.-22263A>G variant was associated with a consistent decrease in luciferase activity, which may result in decreased RF metabolism and increased plasma RF concentration [74]. However, Dompreh et al. did not find any changes in the RF exposures with the *CES2* rs3759994 variant (Table 5) [43].

## 6. AADAC

AADAC is an enzyme expressed primarily in the human liver and intestine that causes the hydrolysis of many drugs [75]. Nakajima et al. reported that human AADAC was the enzyme responsible for the deacetylation of RF to 25-deacetylrifampin [15]. The *AADAC* rs1803155 and rs61733693 variants which are missense mutations did not affect any changes in the plasma RF concentrations (Table 6) [55].

## 7. Vitamin D Pathway Gene Polymorphisms

The Caudal-type homeobox protein 2 (*Cdx2*) gene variant found in the regulatory region, *FokI* variant in exon 2 and *BsmI*, *TaqI* and *ApaI* variants in the 3′end of the *VDR* gene were found to be associated with TB [76]. *BsmI* (rs1544410), *FokI* (rs10735810), *TaqI* (rs731236) and *ApaI* (rs7975232) represent the most commonly occurring SNPs of *VDR* gene [77]. At the fourth week of ATT, univariate regression analysis revealed that *FokI* TC/CC genotype had a negative predictor role on the plasma RF C_trough_ (*p* = 0.694, β = −0.085, 95% CI [-1314.809-891.285]), possibly due to stronger transcription of the RF influx protein [38]. The *FokI* variant codes for a shorter 424 amino acid VDR protein isoform which shows a comparatively higher transcriptional activity by displaying enhanced interaction with transcription factor IIB [78]. Recently, Shaik et al. reported the frequencies of *FokI* TT, TC and CC genotypes to be 30.2%, 34.4% and 27.7%, respectively, in the Saudi Arabian population [79]. Reports from the Brazilian population revealed the frequencies of *FokI* TT, TC and CC genotypes to be 44.6%, 41.4% and 14%, respectively [80]. Calcagno et al. reported that the *VDR* regulatory region *Cdx2* variant was not associated with any significant changes in the plasma RF concentration [52].

CYP27B1 and CYP24A1are two enzymes that are involved in the biotransformation of Vitamin D and play critical roles in governing the 1α,25-dihydroxyvitamin D_3_ (1,25-(OH)_2_D_3_) concentration. *CYP27B1* gene encodes for the 1α-hydroxylase enzyme that is involved in the activation of 25-hydroxyvitamin D_3_ (25-OH-D_3_) to 1,25-(OH)_2_D_3_ [81]. *CYP24A1* is involved in catalyzing the C-23 and C-24 hydroxylation pathways of 25-OH-D_3_ and 1,25-(OH)_2_D_3_ [82]. Hence, genetic variants of these genes may alter the Vitamin D levels and may thereby render TB susceptibility as well as alter RF concentrations in plasma. Allegra et al. reported that the *CYP24A1* rs927650 and *CYP27B1* rs4646536 variants increased plasma RF concentrations which may probably be explained by the increased activation of Vitamin D, resulting in reduced RF elimination (Table 7) [38]. Multivariate linear regression analysis revealed that *CYP27B1* rs4646536 variant (+ 2838C>T; CC/CT genotype) located at intron 6 was a positive factor for RF C_max_ concentration ((*p* = 0.024, β = 0.416, 95% CI [469.172–5857.279]) at second week of ATT. Univariate linear regression analysis revealed that for the *CYP24A1* rs927650 (22776C>T) variant located at intron 11, the homologous mutant profile (TT) is a positive predictor factor of RF C_trough_ ((*p* = 0.924, β = −0.021, 95% CI [-1148.256-1055.303]) at fourth week of ATT [38]. The distribution of *CYP27B1* rs4646536 TT, TC and CC genotypes were reported to be 45.7%, 40.4% and 13.9%, respectively, in healthy controls of Germany which were in near similar lines with a previously conducted study among 7435 healthy controls of UK [83,84]. The clear functional status of *CYP27B1* rs4646536 is unknown. Intronic variants could influence gene expression by affecting the binding of transcription factors and mRNA splicing [85,86]. Hence, an allele variation of rs4646536 from C to T can cause abnormal expression of *CYP27B1*, resulting in the alteration of Vitamin D levels. *CYP27B1* rs4646536 was associated with Vitamin D levels and Vitamin-D-related diseases [84]. The frequencies of *CYP24A1* rs927650 TT, CT and CC genotypes were reported to be 26.1%, 49.7% and 24.2%, respectively, in the healthy controls of Germany and 21.3%, 50.8% and 27.9%, respectively, among type 1 diabetes German patients [83,87]. 1α,25(OH)_2_D_3_ exhibit genomic actions that are mediated through the ligand-binding to the VDR, which forms a heterodimer with retinoid x receptor alpha (RXRα) and subsequently binds to Vitamin D response elements (VDRE) to either enhance or repress transcription of various genes [88]. The *CYP24A1* gene has a significant role in 1,25(OH)_2_D_3_ signaling as the promoter region of the *CYP24A1* gene contains VDRE [89]. Polymorphisms in a VDRE of the *CYP24A1* gene could reduce the receptor protein binding, transactivation and expression of the *CYP24A1* gene in vivo [90]. A suggestive relationship between the *CYP24A1* SNP rs927650 and concentrations of 25(OH)D was reported by Hibler et al. [91]. Further research investigating the influence of *CYP27B1* and *CYP24A1* variants on Vitamin D levels and consequently on RF exposures are required to establish conclusive evidence.

## 8. Conclusions

Pharmacokinetic heterogeneity in RF levels represents an austere and ubiquitous problem in TB patient care. This can lead to therapeutic inefficacy, resistance, adverse drug events and increased healthcare expenditures. Genetic variants of *SLCO1B1*, *ABCB1* and *VDR* have attracted scientific attention for their influence on the pharmacokinetics of a wide range of drugs. While there is a vast number of studies that have explored the influence of SNPs with Isoniazid levels in plasma, only a limited number of studies have explored the influence of genetic variants on the RF pharmacokinetics. Evidence available to date reported a lack of any association of *ABCB1*, *PXR*, *CAR*, *CES1* and *AADAC* genetic variants with the RF concentrations in plasma. Some literature has shown an association of certain genetic variants of *SLCO1B1*, *CES2* and Vitamin D pathway genes with significant variations of RF concentration in plasma. A comprehensive exploration of the role of genetic variants of these genes can be initiated to provide a consensus agreement on their influence on RF pharmacokinetics in different populations.

Genotyping offers to be a potential tool of precision medicine for predicting individual drug-metabolizing and drug transport capabilities before initiation of RF treatment. Further studies assessing RF exposure and correlating it with the genetic polymorphisms are required in different ethnic populations. Besides, such research should be based on a representative and appropriate sample size to validate and implement a cost-effective genotyping-based RF dosage optimization in clinical settings and national policy levels.

## Figures and Tables

**Table 1:** Influence of *SLCO1B1* genetic variants on plasma RF levels.

Sl No.	Author, Year	Population	SNP ID	Criteria of Sample Collection	Time of Sample Collection	RF Concentration in Plasma
1	Mukonzo et al., 2020 [48]	50 TB patients from Uganda	rs4149056	After 21 days of ATT initiation	Predose, 1, 2, 4, 6 and 12 h postdose	No change
			rs2306283			No change
			rs4149032			No change
2	Naidoo et al., 2019 [51]	172 recurrent TB patients in South Africa	rs2306283	1 and/or 2 months and at 6 months during ATT	Predose, 2.5, 6 and 24 h postdose	No change
			rs4149032			No change
			rs4149056			No change
			rs4149015			No change
3	Calcagno et al., 2019 [52]	221 PTB with HIV patients in Uganda	rs4149032	At 2nd, 4th and 8th week of ATT	1, 2 and 4 h postdose	No change
4	Dompreh et al., 2018 [43]	113 pediatric TB patients in Ghana	rs2306283	After 4 weeks of ATT	Predose, 1, 2, 4 and 8 h postdose	Decreased
			rs11045819			No change
			rs4149056			No change
			rs4149032			No change
5	Allegra et al., 2017 [38]	24 TB patients in Italy	rs4149056	At 2nd week and 4th week of ATT	Plasma C_max_ (end of 3 infusions for IV route and 2 h postdose for oral) and C_trough_	Increased
6	Sloan et al., 2017 [55]	174 adult PTB patients in Malawi	rs11045819	Day 14 or 21 of ATT	Predose, 2 and 6 h postdose	No change
			rs4149032			No change
7	Ramesh et al., 2016 [53]	256 South Indian adult PTB/EPTB patients	rs11045819	After a minimum of 2 weeks of ATT	2 h postdose	No change
			rs4149032			No change
			rs4149033			No change
8	Jeremiah et al., 2014 [54]	PTB patients in Tanzania	rs4149032	1st occasion: 7 ± 2 days after ATT 2nd occasion: Around 56 days after ATT	2, 4 and 6 h postdose	No change
9	Gengiah et al., 2014 [47]	57 TB with HIV patients in South Africa	rs4149032	At 4th, 8th and 12th week of TB treatment	2.5 h postdose	Decreased
10	Chigutsa et al., 2011 [46]	60 PTB patients in South Africa	rs4149032	At least 1 month after the start of ATT	4 to 8 samples per patient, randomly collected over a 7 h period	Decreased
			rs4149056			No change
			rs11045819			No change
11	Weiner et al., 2010 [49]	72 TB Patients (37 from Africa and 35 from the United States and Spain)	rs4149015	Between the 9th and 40th doses in TB patients	Just before dose and 1, 2, 6, 8 to 10, 11 to 13 and 23 to 25 h after dose	No change
			rs2306283			No change
			rs11045819			Decreased
			rs4149056			No change
			rs59502379			No change

**Table 2:** Influence of *ABCB1* genetic variants on plasma RF levels.

Sl No.	Author, Year	Population	SNP ID	Criteria of Sample Collection	Time of Sample Collection	RF Concentration in Plasma
1	Naidoo et al., 2019 [51]	172 recurrent TB patients in South Africa	rs10276036	1 and/or 2 months and at 6 months during ATT	Predose, 2.5, 6 and 24 h postdose	No change
			rs1128503			No change
			rs2032582			No change
			rs1045642			No change
			rs2235033			No change
			rs2235013			No change
2	Calcagno et al., 2019 [52]	221 PTB with HIV patients in Uganda	rs1045642	At 2nd, 4th and 8th week of ATT	1, 2 and 4 h postdose	No change
3	Allegra et al., 2017 [38]	24 TB patients in Italy	rs1045642	At 2nd week and 4th week of ATT	Plasma C_max_ (end of 3 infusions for IV route and 2 h postdose for oral) and C_trough_	No change
4	Chigutsa et al., 2011 [46]	60 PTB patients in South Africa	rs1045642	At least 1 month after the start of ATT	4 to 8 samples per patient, randomly collected over a 7 h period	No change
			rs2032582			
			rs1128503			
			rs3842			

**Table 3:** Influence of *PXR* genetic variants on plasma RF levels.

Sl No.	Author, Year	Population	SNP ID	Criteria of Sample Collection	Time of Sample Collection	RF Concentration in Plasma
1	Naidoo et al., 2019 [51]	172 recurrent TB patients in South Africa	rs2472677	1 and/or 2 months and at 6 months during ATT	Predose, 2.5, 6 and 24 h postdose	No change
			rs1523130			No change
2	Calcagno et al., 2019 [52]	221 PTB with HIV patients in Uganda	rs2472677	At 2nd, 4th and 8th week of ATT	1, 2 and 4 h postdose	No change
3	Allegra et al., 2017 [38]	24 TB patients in Italy	rs2472677	At 2nd week and 4th week of ATT	Plasma C_max_ (end of 3 infusions for IV route and 2 h postdose for oral) and C_trough_	No change
4	Chigutsa et al., 2011 [46]	60 PTB patients in South Africa	rs2472677	At least 1 month after the start of ATT	4 to 8 samples per patient, randomly collected over a 7 h period	No change
			rs1523130			No change

**Table 4:** Influence of *CAR* genetic variants on plasma RF levels.

Sl No.	Author, Year	Population	SNP ID	Criteria of Sample Collection	Time of Sample Collection	RF Concentration in Plasma
1	Chigutsa et al., 2011 [46]	60 PTB patients in South Africa	rs2307424	At least 1 month after the start of ATT	4 to 8 samples per patient, randomly collected over a 7 h period	No change

**Table 5:** Influence of *CES1* and *CES2* genetic variants on plasma RF levels.

CES1						
Sl No.	Author, Year	Population	SNP ID/ Nucleotide Change	Criteria of Sample Collection	Time of Sample Collection	RF Concentration in Plasma
1	Sloan et al., 2017 [55]	174 Adult PTB patients in Malawi	rs12149368	Day 14 or 21 of ATT	Predose, 2 and 6 h postdose	No change
**CES2**						
1	Dompreh et al., 2018 [43]	113 Pediatric TB patients in Ghana	rs3759994	After 4 weeks of ATT	Predose, 1, 2, 4 and 8 h postdose	No change
2	Song et al., 2013 [74]	35 TB patients in South Korea	c.-2548C>T	-	2 h postdose	No change
			c.-2263A>G			Increased
			c.269-965A>G			No change
			c.474-152T>C			No change
			c.615+120G>A			No change
			c.1612+136G>A			No change
			c.1613-87G>A			No change
			c.1872*69A>G			No change
			c.1872*302_304delGAA			No change
			c.1872*445C>T			No change

**Table 6:** Influence of *AADAC* genetic variants on plasma RF levels.

Sl No.	Author, Year	Population	SNPs Investigated	Criteria of Sample Collection	Time of Sample Collection	RF Concentration in Plasma
1	Sloan et al., 2017 [55]	174 Adult PTB patients in Malawi	rs1803155	Day 14 or 21 of ATT	Predose, 2 and 6 h postdose	No change
			rs61733693			

**Table 7:** Influence of *VDR*, *CYP24A1* and *CYP27B1* genetic variants on plasma RF levels.

Sl No.	Author, Year	Population	Gene	SNP ID	Pharmacokinetic Sampling	Sample Timing	RF Concentration in Plasma
1	Calcagno et al., 2019 [52]	221 PTB with HIV patients in Uganda	*VDR*	rs11568820 (*Cdx2*)	At 2nd, 4th and 8th week of ATT	1, 2 and 4 h postdose	No change
2	Allegra et al., 2017 [38]	24 TB patients in Italy	*VDR*	rs731236 (*TaqI*)	At 2nd week and 4th week of ATT	Plasma C_max_ (end of 3 infusions for IV route and 2 h postdose for oral) and C_trough_	No change
				rs10735810 (*FokI*)			Decreased
				rs1544410 (*BsmI*)			No change
				rs11568820 (*Cdx2*)			No change
				rs7975232 (*ApaI*)			No change
			*CYP24A1*	rs927650			Increased
				rs2248359			No change
				rs2585428			No change
			*CYP27B11*	rs4646536			Increased
				rs10877012			No change
